# Unraveling *BRAF* alterations: molecular insights to circumvent therapeutic resistance across cancer types

**DOI:** 10.20517/cdr.2024.213

**Published:** 2025-03-24

**Authors:** Caterina Perfetto, Marianna Aprile, Simona Cataldi, Elisa Giovannetti, Valerio Costa

**Affiliations:** ^1^Institute of Genetics and Biophysics (IGB), National Research Council of Italy (CNR), Naples 80131, Italy.; ^2^Department of Environmental, Biological and Pharmaceutical Sciences and Technologies (DiSTABiF), University of Campania “Luigi Vanvitelli”, Caserta 81100, Italy.; ^3^Department of Medical Oncology, Amsterdam UMC, VU University, Cancer Center Amsterdam, Amsterdam 1081 HV, The Netherlands.; ^4^Fondazione Pisana per La Scienza, San Giuliano Terme 56017, Italy.; ^#^Authors contributed equally.

**Keywords:** BRAF-mutated tumors, drug resistance, targeted therapy, genomic profiling, mutation co-occurrence, mutually exclusive mutations

## Abstract

**Aim:** As intrinsic resistance - often driven by concurrent genomic alterations in tumor suppressor genes or oncogenes - remains a major challenge in oncology, this work aimed to comprehensively analyze *BRAF* somatic alterations across cancer types and identify new potential therapeutic strategies to overcome drug resistance.

**Methods:** We conducted an extensive analysis of genomics, transcriptomics, and clinical data retrieved from public repositories, including cBioPortal. Our comprehensive analysis examined *BRAF* alterations [point mutations, structural variants (SVs) and copy number alteration] in more than 217,000 tumor samples across 120 distinct tumor types from primary and metastatic sites in both adult and pediatric cohorts, focusing on mutual exclusivity and co-occurrence of mutations in other oncogenes or tumor suppressors. The work also explores the association of *BRAF* somatic alterations with survival, clinical and molecular features.

**Results:** Analysis of mutation frequencies across cancer types revealed that BRAFV600E represents approximately 90% of all *BRAF* alterations. While melanoma and thyroid carcinoma show the highest prevalence of *BRAF* mutations, followed by colorectal and non-small cell lung cancer in terms of absolute number of patients harboring *BRAF* mutations worldwide, notably high mutation frequencies were identified in rare malignancies, including hairy-cell leukemia, ganglioglioma, and serous borderline ovarian tumors. The comprehensive analysis of genomic profiling data across these tumors uncovered distinct patterns of co-occurring and mutually exclusive alterations in oncogenes and tumor suppressor genes, illuminating resistance mechanisms and suggesting novel therapeutic combinations.

**Conclusion:** Comprehensive genomic profiling is critical for optimizing targeted therapy and overcoming drug resistance in *BRAF*-mutated cancers. The identification of co-occurring alterations provides opportunities for rational combination therapies, emphasizing the importance of detailed mutation profiling in developing effective treatment strategies across diverse cancer types.

## INTRODUCTION

The genomic profile of tumors significantly influences their response to treatment and the potential to develop drug resistance. Among the most studied oncogenic drivers are *BRAF* mutations, which play a pivotal role in cancer development and in the choice of therapeutic strategies. The *BRAF* gene encodes a serine/threonine kinase, B-raf, that is integral to the MAPK/ERK signaling pathway, a critical regulator of cellular proliferation and survival. The most common mutation, BRAFV600E (c.1799T>A), accounts for approximately 90% of *BRAF* mutations across cancer types^[1]^. This mutation leads to the constitutive activation of B-raf kinase, resulting in enhanced ERK signaling, increased cell proliferation, resistance to apoptosis, and remodeling of the tumor microenvironment^[2,3]^, as well as metabolic reprogramming^[4-7]^. While the prevalence and impact of *BRAF* mutations vary among cancer types, their presence is often associated with poor prognosis^[8,9]^. Therapeutically, *BRAF* mutations are classified into three categories (class I to III) according to their impact on the kinase activity and their sensitivity to B-raf inhibitors (BRAFi)^[10,11]^. Such distinctions underscore the importance of detailed genomic profiling to guide the selection of appropriate therapies^[12]^.

Despite advances in targeted treatments, intrinsic resistance to BRAFi remains a common challenge, often driven by pre-existing factors such as the upregulation of receptor tyrosine kinases (RTKs), PTEN loss, or the presence of a pro-angiogenic tumor microenvironment. Concurrent genomic alterations further complicate treatment outcomes. For example, PTEN loss, occurring in approximately 30% of *BRAF*-mutant melanomas, activates the PI3K/AKT pathway, providing an alternative survival mechanism that circumvents B-raf inhibition^[13]^. Effective management often requires combination therapies targeting both the MAPK and PI3K pathways. Similarly, *TP53* mutations, observed in about 20% of *BRAF*-mutant tumors, compromise cellular stress responses and apoptosis, correlating with more aggressive disease phenotypes and reduced responsiveness to targeted and immunotherapy approaches^[14]^. The genomic context of *BRAF* mutations and the associated response to BRAFi also vary by tumor type. In colorectal cancer (CRC), for instance, *PIK3CA* mutations frequently co-occur with *BRAF* mutations and contribute to primary BRAFi resistance^[15]^. Additionally, intratumoral heterogeneity, where distinct clones harbor either *BRAF* or *RAS* mutations, can drive resistance through divergent evolutionary pressures. Although *RAS* and *BRAF* mutations are typically mutually exclusive within a single cell, their coexistence in separate tumor clones presents significant therapeutic challenges^[16]^. Moreover, alterations in downstream components of the MAPK pathway, such as MEK or ERK, can also bypass B-raf inhibition, while mutations in pathway regulators further modify treatment response. These adaptive mechanisms require regular genomic monitoring to identify resistance as it emerges and to enable adjustments to the therapeutic strategies accordingly. The temporal evolution of the mutational landscape, from primary driver mutations to therapy-induced alterations, further highlights the need for dynamic reassessment of tumor profiles throughout the treatment.

However, the precise genomic characterization of the initial oncogenic driver lesions remains paramount for an optimal therapeutic intervention, superseding temporal monitoring of clonal dynamics, as it enables targeted treatment selection while minimizing the probability of acquired resistance mechanisms. Advances in understanding the interplay of concurrent mutations have led to the development of more refined therapeutic strategies, often involving combination regimens tailored to the tumor’s specific genomic landscape.

In this *scenario*, our study provides a systematic analysis of somatic *BRAF* alterations across diverse cancer types. Particular emphasis is given to rare *BRAF*-driven malignancies, such as CRC and non-small cell lung carcinoma (NSCLC), which exhibit unsatisfactory responses to BRAFi compared to melanoma and thyroid carcinomas. Indeed, in these latter malignancies, therapies based on BRAFi - alone or in combination with MEKi - are already approved (although for metastatic and aggressive cases) and effective, whereas only preclinical and initial clinical attempts have been made for the other cancer types. Our analysis in multiple *BRAF*-mutated tumors proposes novel therapeutic modalities utilizing BRAFi in conjunction with agents targeting orthogonal, MAPK-independent pathways. This systematic work underscores the clinical significance of accurate *BRAF* mutation profiling, as distinct alterations demonstrate variable therapeutic responses across distinct cancer subtypes. Future intervention strategies cannot ignore a comprehensive genomic characterization to facilitate the definition of rational drug combinatorial approaches for enhanced therapeutic efficacy.

## METHODS

### Selection of genomic data set of tumor samples

Genomic data sets available at the cBioPortal repository (https://www.cbioportal.org; accessed from January to April 2023)^[17-19]^ were systematically screened for a total of 217.491 tumor samples. The analysis was performed on two distinct, large and independent patient cohorts, namely cohort #1 and cohort #2. The former, consisting of 65,970 patients (from 69.340 distinct samples), was created using the tab “Curated set on non-redundant studies” at cBioPortal and includes the combination of 214 independent (TCGA and non-TCGA) studies with no overlapping samples, encompassing about 100 distinct tumor types from primary and metastatic tumor sites in adult and pediatric groups [Supplementary File 1]. The latter cohort derives from the AACR Project GENIE (Genomics Evidence Neoplasia Information Exchange) v13.0-public (available on the cBioPortal repository), consisting of 148.268 patients (with 167.423 analyzed tumor samples), covering more than 110 different tumor types [Supplementary File 1].

### Analysis of mutation frequencies, mutual exclusivity and co-occurrence

**Figure 1:**
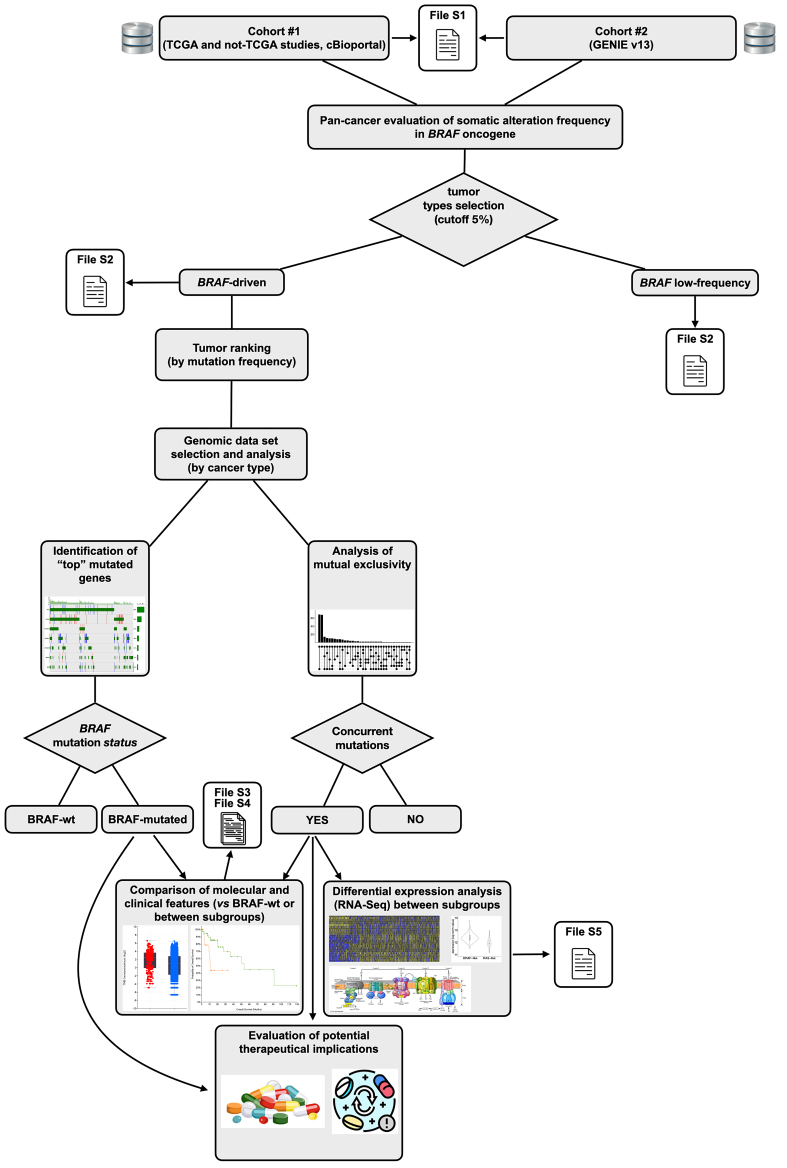
Workflow of pan-cancer analysis of *BRAF* alterations across tumor types. Workflow of the analysis on the genomic data sets to evaluate - across multiple cancer types - the frequency of *BRAF* alterations (point mutations, deletions/amplifications and structural variants), associations with molecular/clinical features and mutational co-occurrence. Tumors were selected on a mutation frequency cutoff of 5% from the TCGA and non-TCGA datasets in cBioPortal. Cohort descriptions are provided in the main text and in the Methods section.

The analysis of the above-mentioned genomic data sets focused on point mutations, gene fusions [structural variants (SVs)], and copy number alterations (CNAs), mainly in the *BRAF* gene. However, where specified for specific purposes, the analysis was extended to other driver mutations in oncogenes with high mutation frequency (top-ranked driver genes for each tumor type were considered). As schematized in Figure 1, tumors were ranked according to their mutation frequency in *BRAF* oncogene and the specific studies (genomic data sets) with very low mutation frequencies (< 5%) were discarded from further analyses. On the contrary, tumor types above the frequency threshold were analyzed. In this case, all the independent studies (for the same tumor type) available on cBioportal with >100 patients were combined together. Then, for each tumor type - independently for cohort #1 and cohort #2 samples - the most frequently mutated genes were identified, and the main representative pathways (e.g., MAPK, PI3K/Akt) were further investigated and graphically reported. Visual representation of the results of this analysis was performed using OncoPrints for the tumors of interest. These graphical illustrations were drawn using the *oncoPrint* function in R language into custom scripts. Based on the OncoPrints, trends in mutual exclusivity or co-occurrence between gene pairs (BRAF and other top-mutated oncogenes or suppressor genes) within a gene set were evaluated. After selecting the gene pairs, systematic analysis to evaluate mutual exclusivity or co-occurrence for each tumor of interest was carried out using the tool implemented in the cBioPortal repository. Where significant, the results are reported (log_2_ odds ratio and *Q*-value) in the related figure, for each tumor type and cohort analyzed.

### Association of *BRAF* mutations with survival, clinical and molecular features

For the top-ranking tumors in the mutational frequency analysis, we stratified patients according to the *BRAF* mutational status (“*BRAF*-altered” and “*BRAF*-unaltered”). In these subgroups, and for each analyzed tumor type, we evaluated the survival and the statistically significant enrichment of specific clinical and molecular features, possibly associated with the presence of *BRAF* mutations, by using the “compare” function implemented in cBioPortal. This analysis was performed for both the cohorts under evaluation. For cohort#1, the clinical/molecular parameters considered were those available for TCGA studies, whereas for cohort#2, those available in the GENIE v13.0-public data. As reported in cBioPortal, for the survival analysis, the log-rank test was used to test the null hypothesis that there is no difference between the groups in the probability of an event at any time point. Hazard ratios were derived from the log-rank test. Survival analysis was performed only on patients from cohort#1, because of the lack of survival data in the GENIE v13.0-public series. For cohort#2 rare tumors, we evaluated the significant association with the “Vital status” parameter. Likewise, because of the completeness of clinical/molecular attributes for cohort#1 samples, we considered only these samples for the association analyses. A *P-*value of the null hypothesis - derived from the chi-squared test or Kruskal-Wallis test - was computed for each pair (*BRAF* status and clinical/molecular attribute); *Q*-values - after Benjamini-Hochberg FDR correction- < 0.05 were considered significant and reported in the Figures. The same analyses were performed considering, for each tumor type analyzed, the different subgroups of patients stratified according to the presence of concurrent mutations (“*BRAF-pure*” *vs*. “*BRAF + other_oncogenes*”).

### Differential expression, gene ontology and pathway analysis

To identify differentially expressed genes between the above-mentioned subgroup pairs, i.e., “*BRAF-altered*” *vs*. “*BRAF-unaltered*” and *“BRAF-pure*” *vs*. “*BRAF + other_oncogenes*” - for each specific tumor as reported in the Results section - were considered only curated data sets from TCGA. Using the “mRNA” and “Protein” tab function of cBioportal, which include mRNA expression data as RSEM (Batch normalized from Illumina HiSeq_RNASeqV2) and protein expression, measured by reverse-phase protein array (RPPA), respectively, we selected only gene lists with Benjamini-Hochberg adjusted *P-*values (*Q-*value < 0.05). Gene Ontology of differentially expressed genes and proteins was evaluated by using the Functional Annotation Tool of DAVID resource^[20,21]^ (https://davidbioinformatics.nih.gov). The analysis of enriched pathways was performed using the KEGG database^[22]^ (https://www.genome.jp/kegg/).

## RESULTS

### Pan-cancer survey on *BRAF* somatic alterations

We screened genomic data sets from a total of 217,491 tumor samples divided into two distinct large and independent patient cohorts [Supplementary File 1]. The cohort #1 consists of 65,970 patients (69.340 samples) encompassing about 100 distinct tumor types from primary and metastatic tumor sites in adult and pediatric cohorts [Supplementary File 1]. This cohort comes from the combination of 214 independent (TCGA and non-TCGA) studies from the manually curated set of the cBioPortal repository (https://www.cbioportal.org). Cohort #2 is the AACR Project GENIE v13.0-public - recently released and available on the cBioPortal repository - consisting of 148.268 patients (with 167.423 analyzed tumor samples), covering more than 120 different tumor types [Supplementary File 1]. The analysis of *BRAF* somatic alterations in these two cohorts revealed that about 5.3%-6.3% of all oncologic patients (3401/63977 profiled for *BRAF* gene in cohort #1, i.e., 5.32%; 9360/148234 profiled for *BRAF* gene in cohort #2, i.e., 6.31%) carry either somatic point mutations, gene fusions (SVs), or CNAs in *BRAF* oncogene [Supplementary File 2]. From a pan-cancer perspective, only a very small fraction (less than 1%-2%) of tumor samples (94/29333 profiled for SVs, i.e., 0.3%, in cohort #1; 498/25792 profiled for SVs, i.e., 1.9%, in cohort #2) carries SVs within *BRAF* oncogene [Supplementary File 2] and only few rare brain tumors, such as the pilocytic astrocytoma (46.7%), low-grade glioma (7%), and ganglioglioma (6.5%), display far higher occurrences of genomic rearrangements in *BRAF*. Likewise, CNAs, and especially amplifications, in *BRAF* are quite rare events across all cancer types (423 on 30,463 profiled for CNAs, i.e., 1.4% in cohort #1; 224 on 117,074 profiled for CNAs, i.e., 0.2%, in cohort #2), except in melanoma (9.5%)^[23]^, serous ovarian cancer [8.5% in cohort #1; Supplementary File 2], urothelial carcinoma (5.5%)^[24]^, and metastatic prostate adenocarcinoma (4.2%-4.9%)^[25,26]^, where their occurrence is noteworthy. Interestingly, despite CNAs in *BRAF* being rare in breast invasive ductal carcinomas (0.5% in cohort #1 and 1.5% in cohort #2), in line with the high incidence of this tumor type, a relevant number of breast cancer patients displays at least one *BRAF* CNA (> 350 patients in the cohort #1 and about 300 in cohort #2). On the contrary, in well-known *BRAF*-driven tumors such as papillary, poorly-differentiated and anaplastic thyroid carcinomas [papillary (PTC), poorly diff (PDTC) and anaplastic (ATC), respectively)], *BRAF* amplifications have never been reported, or at least are not reported in the above-mentioned databases.

The most frequent *BRAF* alterations are somatic point mutations (in the protein-coding region), which occur in about 85%-97% of all *BRAF*-mutated tumor patients (2878/3401 *BRAF*-mutated samples in cohort #1, i.e., 84.7%; 9053/9360 *BRAF*-mutated samples in cohort #2, i.e., 96.7%). In particular, the valine at position 600 (in the exon 15) is the most frequently mutated B-raf protein residue, and the most common change is BRAFV600E - which is the mutational hotspot in (almost) all tumor types (about 45%-50%). Our analysis revealed that, despite the mean mutation frequency across all tumors analyzed in both cohorts being about 5%-6%, PTC (57% in cohort #1 and 63% in cohort #2) and ATC (45% in cohort #1 and 38% in cohort #2), as well as melanoma (45%-49% in cohort #1 and 35%-42% in cohort #2), display far higher *BRAF* mutation frequencies [Supplementary File 2]. Moreover, a remarkably high *BRAF* mutation frequency (about 95%) was observed in a cohort #1 study on metastatic melanoma^[27]^. The interrogation of genomic data sets of some rare tumor subtypes - hairy-cell leukemia, ganglioglioma, and serous borderline ovarian tumor (in cohort #2) - revealed very high frequencies of *BRAF* mutations in these tumors (69%, 53%, and 36%, respectively). We noticed that 98% (58/59), 79% (70/89), and 63% (19/30) of *BRAF*-mutated hairy-cell leukemia, ganglioglioma, and serous borderline ovarian cancer patients, respectively, carry BRAFV600E mutation [Supplementary File 2]. Conversely, despite the relatively low *BRAF* mutation frequency in CRC (11%-13% in cohort #1 and 9%-13% in cohort #2) and NSCLC (3%-5% in cohort #1 and 5%-6% in cohort #2) - being much lower than the one observed in melanoma, PTC, and ATC - because of their high incidence (i.e., these tumors account for more than 23% of all cancer cases), a huge number of patients suffering from these tumors carry *BRAF* gene alterations [Supplementary File 2]. Indeed, considering the overall *BRAF* mutation count (i.e., the number of patients carrying *BRAF* somatic point mutations), CRC and NSCLC rank 2nd and 3rd after melanoma. Therefore, in the following steps, we focused our analyses on these two very common tumors as well as on the above-mentioned rare *BRAF*-mutated malignancies.

### Association of *BRAF* mutations with molecular and clinical outcomes

To investigate the potential clinical implications of *BRAF* mutations in various cancer types, and potentially use this information to inform treatment decisions, we stratified samples according to the *BRAF* mutation *status* in “*BRAF*-mutated” and “*BRAF*-unaltered”. In particular, given the high frequency and the drug targetability, we focused on point somatic mutations, including, in the former group, patients with all point somatic mutations (not only *BRAFV600E*) in the *BRAF* oncogene.

**Figure 2:**
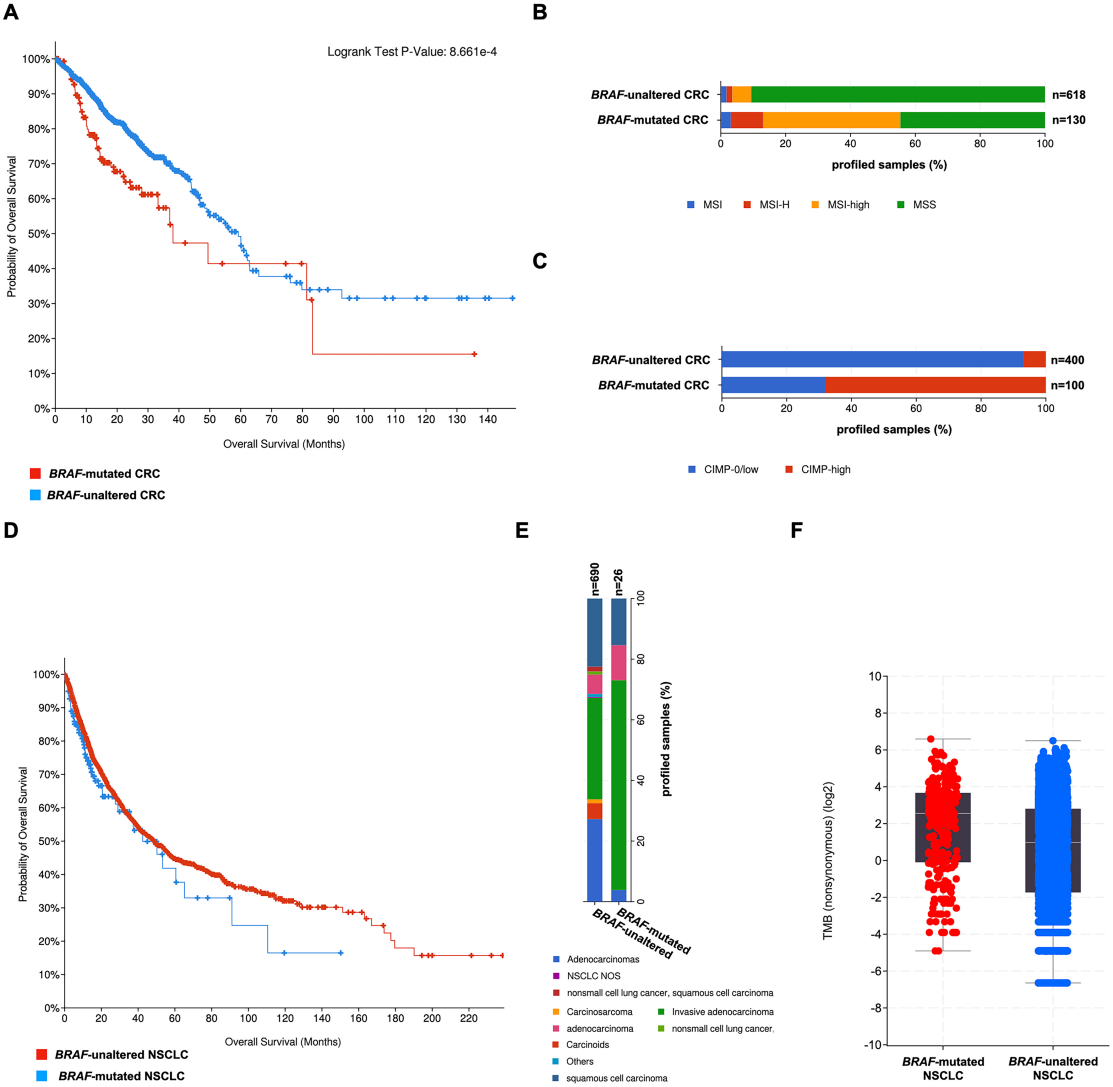
Association analysis of *BRAF* mutations with molecular and clinical outcomes in CRC and NSCLC. (A) OS in CRC patients carrying (red line) or not (blue line) *BRAF* mutations on Kaplan-Meier graphs; (B) Patterns of MSI and (C) CIMP in *BRAF*-mutated and -unaltered CRCs; (D) OS in NSCLC patients carrying (blue line) or not (red line) *BRAF* mutations on Kaplan-Meier graphs; (E) Incidence of specific tumor forms and (F) TMB in *BRAF*-mutated and -unaltered NSCLCs. CRC: Colorectal cancer; NSCLC: non-small cell lung carcinoma; OS: overall survival; MSI: microsatellite instability; CIMP: CpG island methylator phenotype; TMB: tumor mutational burden; MSS: microsatellite stable.

Unfortunately, we did not observe significant changes in the clinical and/or in survival data associated with *BRAF* alterations in the above-mentioned rare tumor subtypes (i.e., hairy-cell leukemia, ganglioglioma. and serous borderline ovarian tumor), possibly because of the very low number of genome-profiled tumor samples available. Conversely, stratifying CRC and NSCLC patients according to the presence of *BRAF* mutation, we disclosed multiple relevant associations [Supplementary Files 3 and 4]. Indeed, in CRC, a significant reduction in the overall survival (OS) was observed in the *BRAF*-mutated subgroup [Figure 2A]. When we also considered molecular outcomes, we found that these tumors also display significantly increased microsatellite instability [MSI; Figure 2B] - associated with low 5-fluorouracil response and predictor of sensitivity to immunotherapy-based treatments^[28]^ - and higher prominence of the CpG island methylator phenotype [CIMP; Figure 2C], in line with the work of Weisenberger *et al.*^[29]^. *BRAF*-mutated CRCs also display increased tumor mutational burden (TMB) and mutation count compared to *BRAF*-unaltered tumors [Supplementary File 3].

The analysis of NSCLC patients did not reveal differences in the KM curves, with no significant variations in the OS of *BRAF*-mutated (*vs. BRAF*-unaltered) NSCLC samples [Figure 2D]. However, we noticed a significant increase in invasive adenocarcinoma forms (70% *vs.* 33%) associated with (i) the presence of *BRAF* alterations [Figure 2E]; (ii) higher TMB [Figure 2F]; and (iii) mutation count for *BRAF*-mutated (*vs. BRAF*-unaltered) tumors [Supplementary File 4]. Interestingly, the analysis also revealed a significant correlation with the “*STK11 mutation status*” - associated with poor survival in metastatic NSCLC^[30]^ - prompting us to further explore, in a systematic way, the possible presence of concurrent mutations in *BRAF*-mutated patients for all the above-mentioned tumors.

### Identification of concurrent mutations in *BRAF*-driven cancers

The analysis of genomic datasets in the most frequently mutated *BRAF*-driven cancers, i.e., PTC, ATC, and melanoma, revealed a peculiar scenario of either mutually exclusive or concurrent mutations. For instance, in PTC patients, except for *TERT* promoter mutations - which display a significant co-occurrence (only in cohort #1 samples) - *BRAF* somatic mutations are mutually exclusive with those in the other tumor driver genes [*H*-, *N*-, and *KRAS* genes and *RET* oncogene; Supplementary Figure 1A]. Likewise, the most aggressive thyroid carcinoma form (i.e., the anaplastic, ATC) displays mutually exclusive mutations for the three most frequently mutated genes [i.e., *BRAF*, *TERT*, and *TP53*; Supplementary Figure 1B]. In melanoma, *BRAF* mutations are mutually exclusive with those in *NRAS* (in both the cohorts) and *NF1* (in cohort #1), whereas they co-occur with *CDKN2A* oncogene alterations [Supplementary Figure 1C]. Hence, except for a very few cases, in cancers where *BRAF* is the main tumor driver gene (i.e., thyroid and skin carcinomas), all *BRAF* somatic alterations are mutually exclusive with those in other driver oncogenes and/or tumor suppressors.

**Figure 3:**
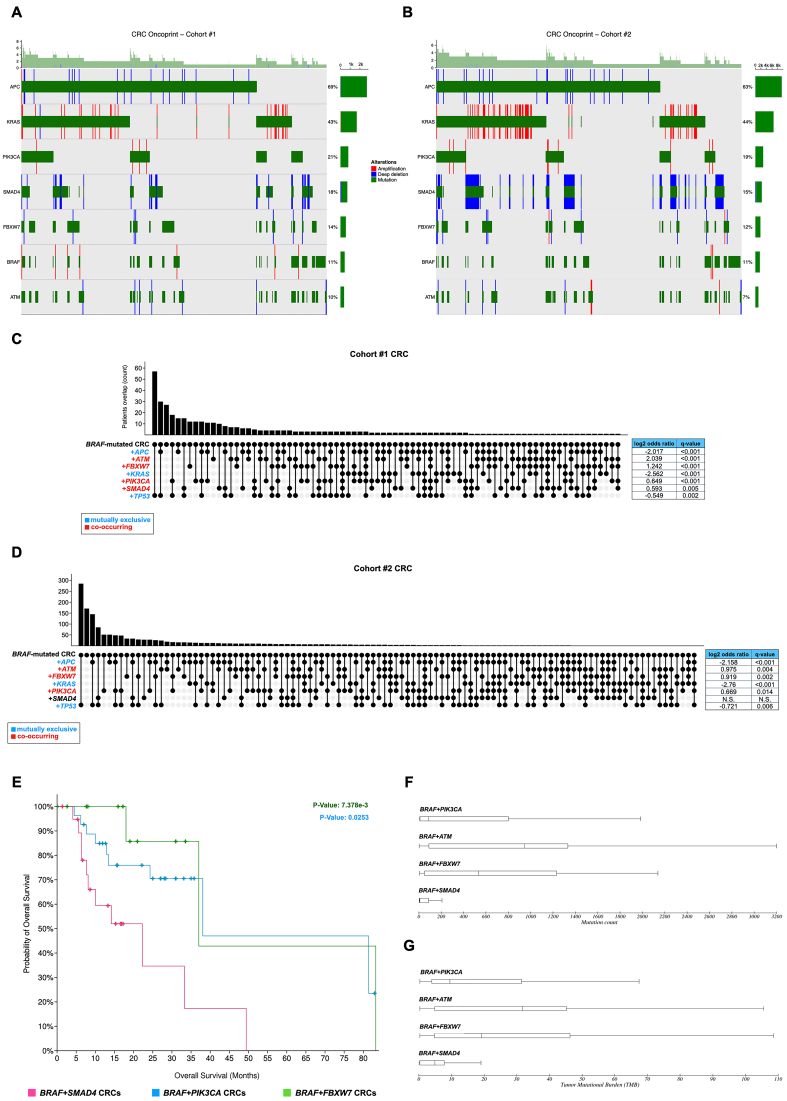
Comparative analysis of gene co-mutation patterns in CRC patients carrying or not *BRAF* mutations. (A and B) Oncoprint of gene mutations (cutoff 5%) indicating co-mutations or mutual exclusivity patterns in *BRAF*-mutant CRC patients of cohort #1 (A) and cohort #2 (B); (C and D) Bar plot of the number of *BRAF*-mutant CRC patients of cohort #1 (C) and cohort #2 (D) with mutational overlap. The spots under the bar plot show the presence of mutually exclusive or co-occurrence mutations (highlighted in blue and red, respectively). On the right, for each mutated gene, the table indicates the association parameters and statistical significance; (E) OS in CRC patients carrying *BRAF* mutations and co-occurrent mutations in *SMAD4* (magenta), *PIK3CA* (blue), and *FBXW7* (green) on Kaplan-Meier graphs; (F and G) Box plots showing mutation count (F) and TMB (G) in CRC patients carrying co-occurrent mutations in *BRAF* oncogene and *ATM, SMAD4*, *PIK3CA* or *FBXW7* genes. CRC: Colorectal cancer; OS: overall survival; TMB: tumor mutational burden; ATM: ataxia-telangiectasia mutated.

To verify whether this genomic feature is also shared with other selected *BRAF*-mutated tumors, we explore the co-occurrence, or mutual exclusivity, of *BRAF* mutations with those in other tumor driver genes. For this analysis, we selected only curated genomic studies (see Methods), and for each tumor type, we analyzed the mutation frequencies in tumor driver genes as well as in those belonging to the most enriched tumor-associated pathways. This analysis revealed that - compared to PTC, ATC, and melanoma - somatic alterations in oncogenes (or tumor suppressors) co-occur with *BRAF* mutations at far higher frequency in these tumors. Indeed, as reported in Figure 3A and B, about 11% of CRC patients (of a total of about 19,000 patients in analyzed samples’ cohorts) harbor somatic *BRAF* mutations, which are mutually exclusive (log_2_ ratio ≤ 0; *Q*-value < 0.05) with those occurring in the three top-mutated CRC genes [i.e., *TP53*, *APC*, and *KRAS*; Figure 3A and B]. These data indicate that - at least in this subgroup of patients having mutually exclusive mutations - the alterations of *BRAF* oncogene represent the main tumor-driving event. More interestingly, *BRAF*-mutated CRCs display a strong pattern of mutation co-occurrence (log_2_ ratio > 0; *Q*-value < 0.05) with *PIK3CA*, *SMAD4*, *FBXW7*, and ataxia-telangiectasia mutated (*ATM*) genes, verified in both the large cohorts [Figure 3C and D], which suggests relevant therapeutic implications. Additionally, CRC patients with concurrent mutations in *BRAF* and *SMAD4* genes display lower OS compared to *BRAF*-mutated CRCs carrying mutations either in *FBXW7* or *PIK3CA* genes [Figure 3E] and lower mutation count and TMB [Figure 3F and G].

Considering that *BRAF*-mutated CRC displays significant enrichment in specific clinical features and/or variation in the OS as well as peculiar patterns of mutational concurrence, we evaluated if they may also display specific gene expression patterns. Hence, we performed multiple comparisons using public transcriptomic (RNA-Seq) data - available for TCGA cohort#1 - of “*BRAF*-altered” *vs*. “*BRAF*-wt” CRC samples. Interestingly, we observed that the two CRC subgroups significantly differ in the expression of genes involved in “positive regulation of transcription from RNA polymerase II promoter” (FDR 2.1E-2), “Wnt signaling” (FDR 8.4E-3), “cell-cell signaling” (FDR 1.0E-3), and “signaling by GPCR” (FDR 8.4E-3). Moreover, “metabolism”, “Wnt signaling”, and “PI3K/Akt” rank as the most affected pathways (KEGG database), together with “Signaling pathways regulating pluripotency of stem cells” [including *OTX1*, *HOXD1*, *NODAL1*, *LEFTYs*, and *WNTs* genes; Supplementary File 5]. The integration of genomics and transcriptomics findings may be relevant to drive specific, and most effective, therapeutic strategies in these tumors (paragraph 3.4).

**Figure 4:**
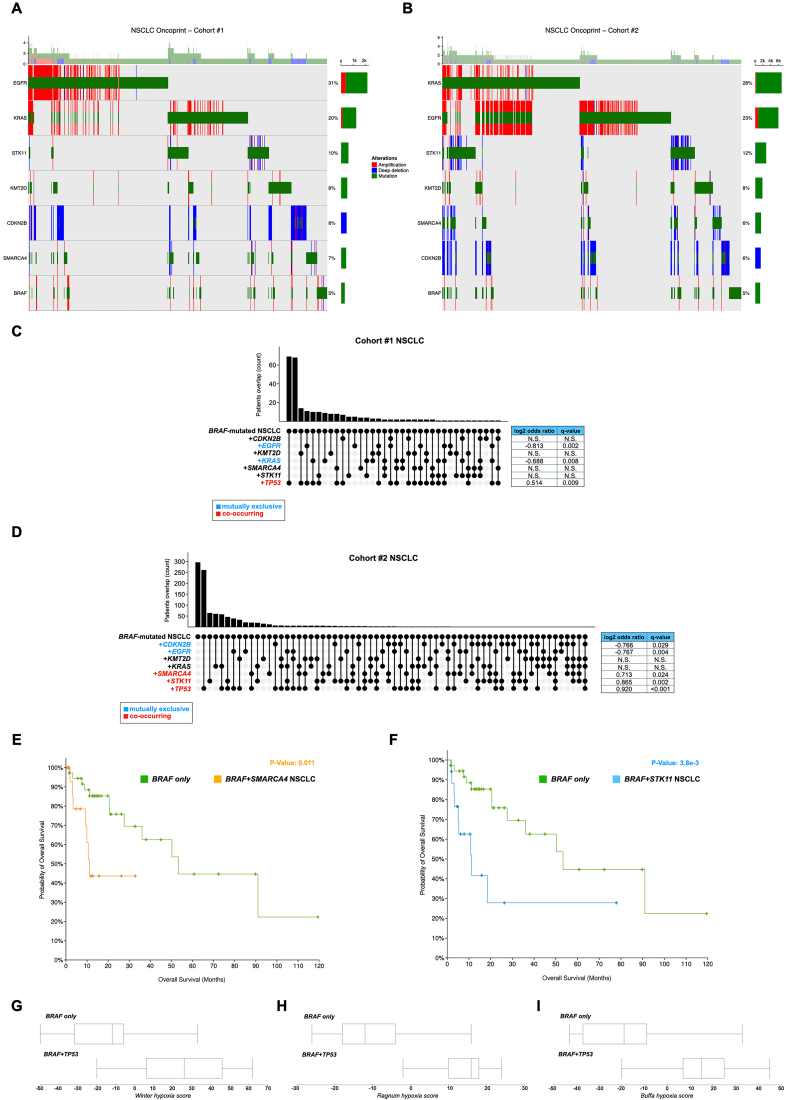
Comparative analysis of gene co-mutation patterns in NSCLC patients carrying or not *BRAF* mutations. (A and B) Matrix-like heatmap (oncoprint) of gene mutations (cutoff 5%) indicating co-occurring or mutual exclusivity mutational patterns in *BRAF*-mutant NSCLC patients of cohort #1 (A) and cohort #2 (B); (C and D) Bar plot of the number of *BRAF*-mutant NSCLC patients of cohort #1 (C) and cohort #2 (D) with mutational overlap. The spots under the bar plot show the presence of mutually exclusive or co-occurrence mutations (highlighted in blue and red, respectively). On the right, for each mutated gene, the table indicates the association parameters and statistical significance; (E and F) OS in NSCLC patients carrying *BRAF* mutation alone (green) or co-occurrence of (E) *SMARCA4* (orange) or (F) *STK11* (blue) mutations, on Kaplan-Meier graphs; (G-I) Box plots showing Winter (G), Ragnum (H), and Buffa (I) hypoxia scores in NSCLC patients carrying *BRAF* mutation alone or co-occurrent with the *p53* gene. NSCLC: Non-small cell lung carcinoma; OS: overall survival.

The same analysis to identify concurrent mutations in NSCLC patients revealed that about 5% of them harbor *BRAF* alterations [Figure 4A and B] and, interestingly, that these mutations are mutually exclusive with those in genes ranking among the most frequently altered in this tumor type [Figure 4C and D], i.e., *EGFR*, *KRAS*, and *CDKN2B*. On the other hand, *BRAF* mutations significantly co-occur with those in *TP53*, *SMARCA4*, and *STK11*. *BRAF*-mutant NSCLC patients with concurrent *STK11* or *SMARCA4* mutations display significantly lower OS compared to patients carrying exclusively mutations in *BRAF* [i.e., “*BRAF* only” subgroup; Figure 4E and F]. Interestingly, as reported in Figure 4G, H, and I, tumors carrying *BRAF* and *TP53* mutations display increased Winter, Ragnum, and Buffa hypoxia scores compared to the “*BRAF* only” subgroup. It is particularly relevant as more than half (58% in cohort #1 and 50.2% in cohort #2) of the *BRAF*-mutated NSCLC harbor *TP53* mutations, which frequently co-occur with mutations in other oncogenes [Figure 4C and D]. As described for CRC, we also performed a comparative transcriptomic analysis in the NSCLC cohort#1 of “*BRAF*-altered” *vs*. “*BRAF*-wt” samples (the LUAD cohort from TCGA). However, unlike CRC, when stratifying NSCLC samples according to the presence of *BRAF* mutations, we could not identify enough differentially expressed genes to perform a robust and statistically significant Gene Ontology and KEGG pathways analysis. This finding further highlights the primary relevance of the somatic genomic background in the choice of the right therapy for *BRAF*-mutated NSCLC.

**Figure 5:**
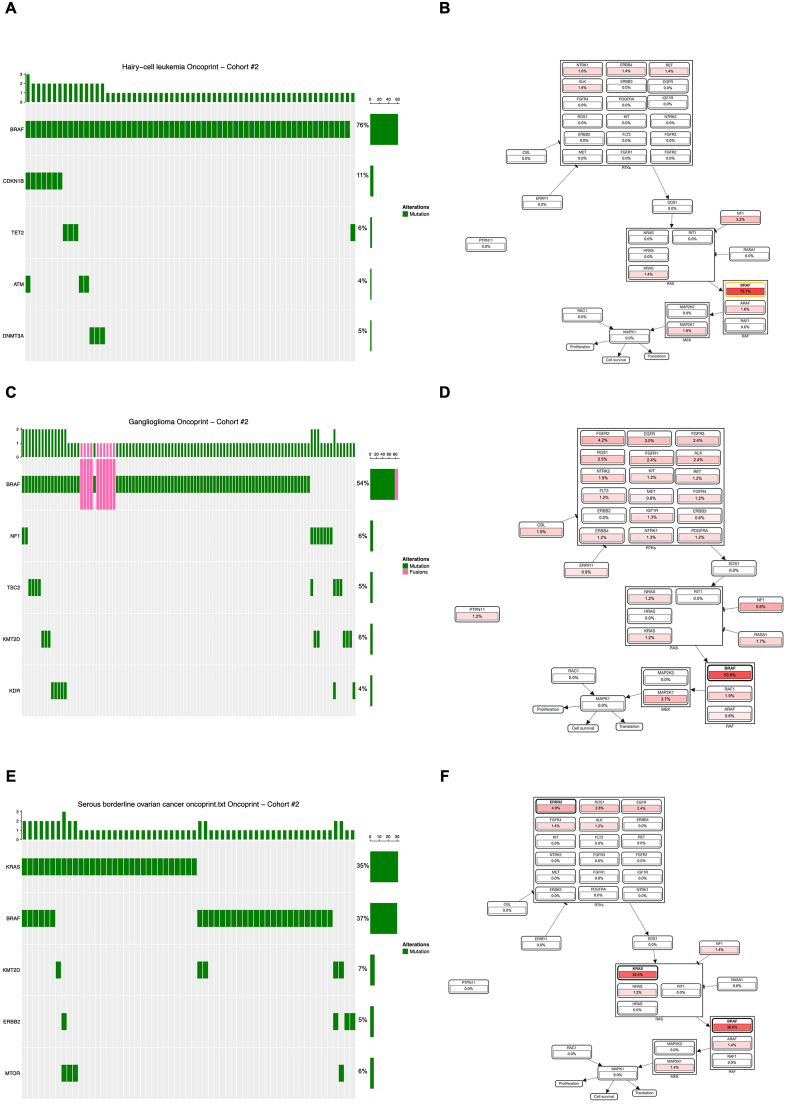
Subtype-specific analysis in *BRAF*-mutated rare tumors. (A and B) Oncoprint displaying gene mutations (cutoff 5%; A) and mutation frequency diagrams in MAPK and RTKs for hairy-cell leukemia (B); (C and D) Oncoprint displaying gene mutations (cutoff 5%; C) and mutation frequency diagram in MAPK and RTKs for ganglioglioma (D); (E and F) Oncoprint displaying gene mutations (cutoff 5%; E) and mutation frequency diagram in MAPK and RTKs for serous borderline ovarian tumor (F). In each box, the red color intensity is proportional to the mutation frequency of the gene. RTKs: Receptor tyrosine kinases; ATM: ataxia-telangiectasia mutated.

Finally, the analysis of *BRAF*-mutated rare tumors revealed that *BRAF* mutations are the main oncogenic lesions in hairy-cell leukemia [Figure 5A] and ganglioglioma [Figure 5B] - which display less frequent mutations in few other oncogenes - whereas in serous borderline ovarian tumor, *RAS* genes play a major tumor-driving role [Figure 5C]. Because of the very low number of available genomic data sets for these rare tumors, the analysis did not reach the threshold of statistical significance, either for mutually exclusive or co-occurring mutations. However, as reported in Figure 5A, patients with *BRAF*-mutated hairy-cell leukemia display frequent concurrent mutations in genes responsible for DNA repair mechanisms and/or epigenetic regulation, such as *CDKN1B*, *TET2*, *ATM*, and *DNMT3B*, with only very low frequencies (> 2%) for other RTKs [e.g., *RET*, *ERBB4*, and *ALK*; Figure 5B].

In contrast, in *BRAF*-driven gangliomas, no evidence of either concurrent or mutually exclusive mutations was observed [Figure 5C]. Interestingly, this tumor type harbors - differently from the other rare tumors analyzed - *BRAF* gene fusions (chimeric transcripts) that may affect the expression of this oncogene in tumor cells. The presence of such rearrangements should be carefully considered in clinical practice as these patients may have lower responses to BRAFi compared to patients carrying point mutations. Moreover, despite *BRAF* being the top-ranked mutated gene in the MAPK signaling pathway, gangliomas also display mutations in various RTKs (*FGFR1*, *FGFR2*, *FGFR3* and *EGFR*) and in MAPK pathway effectors, such as *NF1* and *MAP2K1* genes [Figure 5D].

Finally, in the serous borderline ovarian tumors, differently from the other two examined rare tumors, and likewise, papillary thyroid carcinoma, *KRAS*, *BRAF*, and *ERBB2* point mutations tend to be mutually exclusive [Figure 5E], with a clear *BRAF*- or *RAS*-driven behavior. Nonetheless, because of the very low number of samples, the analysis of mutual exclusivity was not statistically significant. In the same tumor type, our analysis revealed that few other RTKs display somatic mutation [e.g., *EGFR* and *FGFR4*; Figure 5F].

### Targeted therapies and rational combinations

In line with the evidence that about 98% of *BRAF*-mutated hairy-cell leukemia patients in our study cohort carry *BRAFV600E* mutation, multiple reports on low-dose vemurafenib^[31,32]^ or of other BRAFi alone or in combination with MEKi^[33]^, clearly indicate relevant opportunities for targeting these tumors with BRAFi. Building on the promising results of these studies, the French Innovative Leukemia Organization has recently proposed new recommendations for the use of BRAFi as a first-line treatment in specific cases and for relapsed/refractory patients with hairy-cell leukemia^[34]^. Likewise, our analysis reveals that about 80% of ganglioglioma patients display BRAFV600E mutation. The long-term efficacy of vemurafenib in ganglioglioma was recently evaluated in the AcSé vemurafenib basket study^[35]^, which reported an objective response rate (ORR) of 66.7%, together with prolonged survival, indicating the potential of BRAFi as a promising therapeutic approach for these tumors. Our analysis also revealed that more than 60% of patients with another rare tumor, the serous borderline ovarian cancer, carry the same mutation in the oncogene *BRAF*. Different initial attempts have been made with BRAFi monotherapy, or with BRAFi + MEKi combinations, in relapsing patients (after 2-3 therapy cycles with other compounds)^[36-39]^. Interestingly, despite platinum-based chemotherapy still being the preferred first-line therapy, the above-mentioned studies on serous borderline and low-grade serous carcinomas reported reliable responses to BRAFi, suggesting the opportunity to routinely test *BRAF* mutations in these tumors and use BRAFi in first-line treatment. Overall, further supporting these findings, the recent phase 2 ROAR basket trial demonstrated that dabrafenib and trametinib combination exhibits tumor-agnostic activity in multiple rare *BRAF*-mutated cancers, including low-grade gliomas and hairy cell leukemia^[40]^.

Shifting focus from rare to common tumors, CRC and NSCLC rank, respectively, as the 2nd and 3rd tumors for the number of patients carrying *BRAF* mutations, representing a relevant clinical question to solve. Given the association between *BRAF* mutation status and specific molecular features, such as MSI and CIMP^[41]^, as well as the presence of concurrent mutations, therapeutic strategies for *BRAF*-mutated patients should consider these factors to optimize targeted combination therapies.

For instance, in CRC patients with MSI-H, immunotherapy with immune checkpoint inhibitors such as pembrolizumab represents a valuable strategy. Indeed, a phase 3 open-label trial involving 307 CRC patients with metastatic MSI-H CRC demonstrated that pembrolizumab provided significantly longer progression-free survival compared to chemotherapy when used as first-line therapy. Notably, this included 25% of patients harboring BRAFV600E mutant tumors^[42]^.

Similarly, tumors with CIMP may respond more effectively to epigenetic therapies or agents aimed at restoring normal DNA methylation. In a preclinical study using BRAFV600E-mutated PDX CRC models, 5-azacitidine reduced DNA methylation but did not fully relieve transcriptional repression due to adaptive polycomb repressive complex (PRC) activity. However, combining 5-azacitidine with EZH2 inhibition enhanced therapeutic efficacy, highlighting the potential of dual targeting of DNA methylation and PRC activity as a treatment strategy for BRAFV600E-mutated CRC^[43]^. In addition, CIMP status has been associated with a favorable response to specific chemotherapeutic agents, emphasizing the importance of epigenetic modifications in guiding treatment stratification^[44]^.

Most recently, the introduction of B-raf and Mek inhibitors has revolutionized the treatment of *BRAF*-mutated cancers, with the combination of these inhibitors demonstrating clinical activity across more than 20 BRAFV600-positive tumor types, including lung cancer^[45]^, as reported in the ROAR, NCI-MATCH, and Study-X2101 trials. Notably, the Food and Drug Administration (FDA) has granted accelerated approval to dabrafenib and trametinib for metastatic solid tumors harboring BRAFV600E mutations, which have progressed following prior treatment and have no satisfactory alternative treatment option. This recent tissue-agnostic approval marks a transformative shift, prioritizing mutational status over the tumor’s tissue of origin (https://www.fda.gov).

The exception for this tissue-agnostic indication is CRC because of known intrinsic resistance mechanisms. However, preclinical studies suggested that the unresponsiveness of CRC to B-raf inhibition was mediated through feedback activation of EGFR and might benefit from combination therapy of B-rag and Egfr inhibitors^[46]^. So, the protocol of the VE-BASKET trial was amended to include an arm evaluating the combination of vemurafenib and cetuximab^[45]^, and this combination successfully demonstrated efficacy, showing a response in CRC patients^[47-49]^. In addition, the combination of encorafenib (BRAFi) and binimetinib (MEKi), in conjunction with cetuximab, is approved for the treatment of patients with metastatic CRC harboring the BRAFV600E mutation, following prior therapy. This approval is based on data from the BEACON CRC phase III trial, which demonstrated improved outcomes in previously treated patients in the metastatic setting compared with standard chemotherapy^[50]^. However, based on the updated analyses of the same trial, encorafenib plus cetuximab improved OS, ORR, and progression-free survival, and this doublet was established as a new standard care regimen for previously treated patients with BRAFV600E metastatic colorectal cancer (mCRC)^[51]^. Recent molecular profiling revealed that tumors with higher immune signatures showed a trend toward improved OS with the encorafenib, binimetinib, and cetuximab combination. Commonly acquired alterations after treatment included *RAS*, *MAP2K1*, and *MET* mutations, particularly in patients with high baseline cell-cycle gene signatures. Lastly, baseline *TP53* mutations were linked to acquired *MET* amplification^[52]^.

Mutations in *BRAF* and *RAS* are generally considered mutually exclusive in CRC^[53]^, although some studies suggest this exclusivity is not absolute^[54]^. Typically, RAS/RAF mutation status is determined from a single tumor sample, often from the primary tumor. However, in the era of precision medicine, rebiopsies from metastatic sites may be necessary to capture the evolving mutation profile of the disease. These analyses can reveal more discordant findings, highlighting the importance of considering tumor heterogeneity in personalized treatment planning. Additionally, the majority of CRCs are microsatellite stable (MSS) and exhibit relatively few recurrent mutations. Comprehensive co-mutation profiling, therefore, requires large patient cohorts and broad genomic coverage, as demonstrated in a recent population-based Swedish study by Nunes *et al*. using whole-genome sequencing (*n* = 819 stage I-IV MSS CRCs)^[55]^. Notably, this study identified *BRAF* p.V600E-*RNF43* co-mutations as being associated with poor overall and recurrence-free survival in locoregional CRC. However, the co-mutation did not confer additional prognostic significance beyond that of *BRAF* p.V600E alone, aligning with the lack of prognostic value for *RNF43* in *BRAF* p.V600E mCRCs not treated with targeted therapies.

However, in CRC, the co-occurrence of mutations in genes such as *PIK3CA*, *SMAD4*, *FBXW7*, and *ATM* carries significant therapeutic implications. In particular, ATM kinase, a key regulator of DNA damage response (DDR), activated by Double-Strand Break, has become a focus of active research in novel anticancer therapies^[56]^. The first selective ATM inhibitors, KU-55933 and KU-60019, effectively sensitized cells to radiation and DNA-damaging agents like etoposide, doxorubicin, and camptothecin but were unsuitable for clinical studies due to their poor bioavailability^[57]^. Conversely, the recently developed ATM inhibitor AZD0156 demonstrates high oral bioavailability. Its combination with SN38, the active metabolite of irinotecan, showed synergistic inhibitory effects in preclinical CRC models^[58]^, leading to an ongoing phase 1 trial (NCT02588105). Notably, drug activity correlated with immunoblotting results, which revealed strong activation of the DDR induced by irinotecan. This activation was mitigated by AZD0156, particularly in specific models, with variability in effects likely due to differences in DDR mutation profiles. Thus, understanding these profiles could help identify patients most likely to benefit from this therapy.

Another promising strategy involves combining ATM and checkpoint kinase 1 (Chk1) inhibitors. This combination induced synergistic lethality at low doses by enhancing the activation of cyclin-dependent kinase 1 through reduced phosphorylation at T14 and Y15^[59]^. The treatment also increased the sub-G1 cell population and levels of phospho-histone H2A.X and TdT-mediated dUTP nick-end labeling-positive cells, indicating apoptosis, and exhibited strong antitumor activity in syngeneic tumor mouse models. These results support the development of therapies targeting the genomic instability of cancer cells in CRC. Additionally, a recent study showed that ATM inhibition activates the cGAS/STING pathway and enhances MHC class I expression in CRC cells, and these effects can be further amplified by radiation^[60]^. Animal studies demonstrated increased T cell infiltration and cytotoxicity, leading to improved survival in ATM-deficient tumors. These findings suggest that ATM mutations may predict clinical benefits from radiotherapy combined with immune checkpoint blockade in CRC patients, offering a molecular rationale for using ATM-targeted therapies in CRC treatment.

In line with the previously mentioned studies on co-occurring aberrations in defective DNA mismatch repair, a recent study identified two distinct mutation signature clusters (MSC) in CRC: one characterized by extensive mutations (MSC-1) and the other by dominant somatic copy number alterations (SCNA). MSC-1, associated with defective DNA mismatch repair, exhibited a higher frequency of mutations, including *BRAF*, *ATM*, and notably, *SMAD4*^[61]^. SMAD4 is a critical downstream modulator of the canonical TGFβ signaling pathway^[62]^, which has traditionally been considered “undruggable” due to its lack of enzymatic activity and extensive interface for protein-protein interactions^[63]^. However, a recent pilot drug screening using multiplexed time-resolved fluorescence resonance energy transfer (TR-FRET) identified gambogic acid and gambogenic acid as potential disruptors of the SMAD4-SMAD3-DNA complex^[64]^. These two compounds, which impair tumor cell motility *in vitro*, represent the first evidence of SMAD4’s potential druggability. Another strategy to target SMAD4 consists of developing inhibitors of dual-specificity protein phosphatase 4 (DUSP4). In CRC, *DUSP4* is highly expressed and promotes *SMAD4* degradation through ubiquitination pathways. By regulating *SMAD4* expression, DUSP4 enhances cell proliferation, migration, and invasion. Thus, inhibiting DUSP4 could potentially reduce SMAD4 degradation and inhibit CRC progression^[65]^. Additionally, given SMAD4’s central role in TGF-β signaling, another approach involves inhibiting the entire TGF-β pathway, developing inhibitors that target TGF-β ligands, receptors, and downstream signaling molecules, indirectly regulating SMAD4 function, and inhibiting tumor cell proliferation, invasion, and immune evasion^[66]^. These findings open new avenues for developing therapeutic strategies targeting SMAD4, which could potentially be combined with BRAFi in *BRAF*-mutated CRC patients. Moreover, since TGF-β signaling is an immunosuppressive regulator in the tumor microenvironment of mCRC, combining these targeted therapies with other treatment modalities, such as immunotherapy, may enhance therapeutic efficacy^[67]^. Similarly, in line with our analysis of the genomic data [Figure 2F], patients with *BRAF*-mutated NSCLC and high TMB may benefit from immunotherapy with immune checkpoint inhibitors combined with BRAFi like dabrafenib. However, the efficacy of immune checkpoint inhibitors in this subgroup remains inconclusive, as some smaller studies have shown limited effectiveness when used as monotherapy, with conflicting findings in the literature^[68-70]^. Furthermore, patients with *BRAF*-mutant NSCLC, along with those harboring *EGFR* and *ALK* aberrations, typically exhibit lower or intermediate TMB and microsatellite stability, which may explain their limited response to immunotherapy^[71]^.

Of note, *EGFR* and *BRAF* mutations are normally mutually exclusive, as the coexistence of *EGFR* and *BRAF* somatic mutations is uncommon in NSCLC patients^[72]^. However, previous studies have reported the presence of *KRAS* and *EGFR* mutations, along with non-V600 BRAF mutations, in NSCLC^[73,74]^, and it would be interesting to explore whether BRAF mutations are the primary or secondary oncogenic drivers in these cases.

Conversely, multiple studies consistently identify *TP53* as the most common co-occurring mutation with *BRAF* in various lung cancer cohorts. Specifically, Qu *et al*. reported TP53 as the most frequent co-mutation in 6 out of 53 patients studied^[75]^. Similarly, Myall *et al*. observed *TP53* as the most common concurrent mutation in 5 out of 8 patients^[76]^, while in the retrospective analysis by Kron *et al*., TP53 was the most frequent co-alteration, found in 89 out of 121 *BRAF*-mutated patients^[77]^. Remarkably, patients with double mutations tend to have poorer OS compared to those with a single BRAF mutation. In particular, co-mutations involving *TP53* and *PI3KA* were associated with a negative prognosis^[75,78]^. Interestingly, a study on melanoma and papillary thyroid carcinoma cells showed that *BRAF* mutations increased the expression of oncomiR-3151 through the SP1/NF-κB complex^[79]^. Knockdown of miR-3151 reduced cell proliferation, induced caspase-3-dependent apoptosis, and enhanced TP53 mRNA and protein levels while promoting its nuclear localization. Mechanistically, miR-3151 directly targeted TP53 and its pathway members, linking *BRAF* mutations to TP53 inactivation, and combined inhibition of B-raf (using vemurafenib) and miR-3151 knockdown amplified the therapeutic effects.

Developing drugs targeting p53 presents significant challenges^[80]^. TP53 mutations are highly heterogeneous, making a one-size-fits-all approach unfeasible^[81]^. The lack of a clear mechanism for protein reactivation and the absence of binding pockets (except in cases like Y220C) complicate drug development. While inhibiting protein function by occupying active sites is straightforward, reactivating protein function through compound binding remains elusive^[80]^. Additionally, resistance to *TP53* mutations, off-target effects, and the potential toxicity from p53 accumulation in normal tissues further complicate drug development. However, as technology advances, previously undruggable targets like KRAS are becoming druggable^[82]^, raising hopes that p53-targeting therapies will also progress. Given the high frequency of *TP53* mutations in cancers, targeting p53 could lead to significant breakthroughs in cancer treatment, including *BRAF*-mutated cancers.

Lastly, we observed that tumors harboring co-occurring *BRAF* and *TP53* mutations exhibit significantly elevated hypoxia scores [Buffa, Winter, and Ragnum; Figure 4G-I], calculated based on RNA expression clustering of hypoxia-regulated genes^[83-85]^. This aligns with reports linking *BRAF* mutations to hypoxia across various tumor types, including CRC, melanoma, and thyroid cancers^[4,86,87]^. Additionally, a complex interplay between hypoxia and p53 signaling pathways has been well-documented^[88,89]^. Tumor-associated mutant p53 proteins often exhibit gain-of-function properties that promote cancer progression through interactions with hypoxia, cancer metabolism, and HIF signaling pathways. Furthermore, p53 levels and activities are regulated by hypoxia and HIF signaling in ways that vary depending on the cell or tissue type, as well as the severity and duration of hypoxia. Given that *BRAF* mutations and hypoxia can be observed in several solid tumors, the simultaneous inhibition of B-raf and hypoxia or cancer metabolism-associated pathways could synergistically impair tumor growth, enhance apoptosis, and reduce therapy resistance by addressing both oncogenic signaling and the tumor microenvironment^[90]^. For instance, in our recent study, we identified a distinct metabolic gene signature characteristic of BRAF-mutated tumors, which exhibit a glycolytic phenotype marked by increased glucose uptake, lactate efflux, and elevated expression of glycolytic genes regulated by HIF-1α^[4]^. Notably, HIF-1α stabilization offsets the inhibitory effects of BRAFi on these genes and tumor cell viability. Interestingly, by targeting metabolic pathways with a combination of BRAFi and diclofenac, we successfully suppressed the glycolytic phenotype and synergistically reduced the viability of thyroid tumor cells.

Such an approach may also improve the efficacy of immunotherapies and other targeted treatments by reprogramming the hypoxic tumor niche and overcoming adaptive resistance mechanisms. Further exploration of combination therapies that integrate BRAFi and agents targeting hypoxia and cancer metabolism is warranted to optimize therapeutic outcomes in solid tumors and to reduce the onset of secondary cancer resistance.

Of note, the findings of the current study provide a novel perspective on circumventing therapeutic resistance, offering new molecular insights into *BRAF* alterations and related pathways. By employing a pan-cancer survey of *BRAF* somatic alterations, we uncovered unique findings on concurrent mutations in *BRAF*-driven cancers, challenging existing paradigms and broadening our understanding of these tumors.

In particular, the varying response patterns to BRAFi across different tumor types and molecular contexts underscore the importance of considering both primary mutations and co-occurring alterations when designing combined therapeutic strategies. While the combination of BRAFi with other agents has been explored in previous studies, our approach not only identifies previously unrecognized targets but also highlights the utility of advanced omics approaches in unraveling complex biological interactions. Furthermore, the demonstrated success of combination therapies in CRC and the promising outcomes in rare tumors such as hairy-cell leukemia and ganglioglioma illustrate the potential to enhance therapeutic efficacy and overcome resistance, addressing critical unmet needs in clinical practice.

Personalized approaches integrating comprehensive molecular profiling can guide rational combination strategies. For instance, combining BRAFi with immunotherapy in high-TMB tumors, targeting hypoxia pathways in *TP53* co-mutated cases, or exploring ATM inhibition in specific molecular contexts represents promising avenues for future research.

By providing a foundation for these “personalized combination regimens”, this work holds significant potential to directly improve patient outcomes, particularly in rare tumor populations where tailored therapies can have the greatest impact. These advances lay the groundwork for further clinical validation of rationale combinations, offering a tangible pathway to expanding treatment options and elevating standards of care.

## DISCUSSION

Our comprehensive screening of *BRAF* alterations across multiple tumor types reveals important insights into the relationship between genetic mutations and therapeutic responses to BRAFi. The study’s findings are particularly relevant given the recent FDA approval of tissue-agnostic treatments targeting BRAFV600E mutations^[40,91-93]^, highlighting the importance of understanding the broader genomic context in which these mutations occur. The systematic description of *BRAF* co-occurring mutations across different tumor types, including rare tumors that have been historically understudied, represents a key point of our work. While *BRAF* mutations are well-established drivers in cancers like thyroid carcinomas (PTC and ATC) and melanoma, our analysis brings attention to both common tumors where *BRAF* mutations coexist with other significant genomic alterations (i.e., CRC and NSCLC) and rare cancers (i.e., hairy-cell leukemia, ganglioglioma, and serous borderline ovarian cancer) where *BRAF* mutations frequently occur. Therefore, we report distinct patterns of *BRAF* mutations in distinct cancer types, analyzing the association of clinical parameters, co-occurring mutations, and new potential therapeutic implications. Indeed, the therapeutic landscape for *BRAF*-mutated tumors varies significantly across cancer types. Notably, in rare tumors like hairy-cell leukemia, low-dose vemurafenib has shown remarkable efficacy, leading to its recommendation as a first-line treatment in specific cases^[31,94]^. Similarly, ganglioglioma patients show strong responses to vemurafenib monotherapy, with a 66.7% ORR^[35]^. However, in common cancers like CRC, intrinsic resistance mechanisms necessitate combination approaches^[95,96]^. The triple combination of encorafenib, binimetinib, and cetuximab has become standard care for BRAFV600E metastatic CRC^[97,98]^, while the dabrafenib/trametinib combination has shown efficacy in NSCLC^[99]^. The differential impact of *BRAF* mutations on survival across tumor types merits careful consideration. Our analysis suggests that the inconsistent correlation between *BRAF* mutations and OS is not due to a lack of causality, but rather reflects the complex genomic landscape of specific tumors. Particularly in highly undifferentiated cancers, the progressive accumulation of multiple genomic alterations over time evolves into genomic instability, which significantly contributes to poor prognosis. It indicates that *BRAF* mutations, in this context, may act as key contributors to tumor progression, even though their individual impact is overshadowed by the influence of co-occurring genetic events. These findings underscore the urgency of developing more specific combined therapeutic strategies to target oncogenic drivers and address tumor plasticity, with the aim of improving outcomes for patients with these aggressive malignancies. Conversely, in the context of rare tumors, the limited availability of survival data combined with the small sample sizes within datasets constrains the identification of potential associations between *BRAF* mutations and OS. These tumors are frequently underrepresented in large-scale studies, thereby diminishing the statistical power required to detect significant patterns or correlations.

However, overall, our findings may have immediate clinical implications for tumor profiling and treatment selection. Indeed, the identification of specific co-mutation patterns (such as *BRAF-SMAD4* in CRC or *BRAF-STK11* in NSCLC) that correlate with poorer outcomes suggests the need for comprehensive genomic profiling beyond single-gene testing. The presence of concurrent mutations also points to potential therapeutic strategies, such as combining BRAFi with ATMi in CRC patients with concurrent *ATM* gene mutations, or considering hypoxia-targeting approaches in tumors with TP53 co-mutations showing elevated hypoxia scores. From a practical standpoint, our analysis supports the implementation of comprehensive genomic profiling in clinical practice. The varying response patterns to BRAFi across different tumor types and molecular contexts emphasize the importance of considering both the primary mutation and co-occurring alterations when selecting therapeutic strategies. This is exemplified by the success of combination approaches in CRC and the promising responses to BRAFi in rare tumors like hairy-cell leukemia and ganglioglioma. Our findings suggest that the future of *BRAF*-directed therapy lies in personalized approaches that consider the full spectrum of genomic alterations. Novel therapeutic strategies, such as combining BRAFi with immunotherapy in high-TMB tumors, targeting hypoxia pathways in TP53 co-mutated cases, or exploring ATM inhibition in specific molecular contexts, represent promising directions for future research. The integration of comprehensive molecular profiling into clinical decision making will be crucial for optimizing treatment outcomes across different tumor types, particularly in cases where *BRAF* mutations are not the sole driving force of oncogenesis. This is especially relevant for rare tumors, where molecular understanding can significantly impact treatment choices and patient outcomes. Despite the use of an extensive dataset of over 217,000 tumor samples, as well as rigorous association with several clinical, genetic, and molecular parameters, being notable strengths of our study, it displays some limitations. Indeed, the reliance on publicly available data - which may not fully represent the global patient population as they predominantly originate from specific geographic regions and healthcare systems - means that our findings may not be directly applicable to specific patient populations, at least without further validation. It potentially introduces selection bias and limits the generalizability of our findings to diverse patient populations worldwide. This limitation is particularly relevant for the rare tumors under investigation (i.e., hairy-cell leukemia, ganglioglioma, and serous borderline ovarian cancer), where the number of available samples is often limited, potentially affecting the statistical significance of the findings. Future studies would benefit from international collaborations and the integration of additional datasets to enhance the statistical significance of findings in rare tumor types.

Furthermore, our study focuses primarily on genomic alterations and their associations with clinical outcomes, but does not thoroughly address the potential adverse effects and toxicities of the proposed therapeutic strategies. The safety profiles of combination therapies, particularly in the context of specific co-mutation patterns, require further investigation through prospective clinical trials. This is especially relevant for novel combination approaches, such as those targeting concurrent mutations in ATM or addressing hypoxia-related pathways.

Moreover, the retrospective nature of our analysis also limits our ability to establish definitive causal relationships between specific molecular patterns and treatment outcomes. While we identified significant associations between co-occurring mutations and clinical outcomes, prospective validation studies are needed to confirm these findings and establish their clinical utility. Additionally, our analysis may not fully capture the dynamic nature of tumor evolution and treatment resistance mechanisms, as the genomic data analyzed represent only single time points in the (often long) disease course. To address these limitations, future research should prioritize the establishment of international consortia to expand the representation of diverse patient populations and rare tumor types in genomic databases. Prospective clinical trials investigating the safety and efficacy of proposed combination therapies are essential, particularly within the specific molecular contexts identified in our study. Additionally, longitudinal studies incorporating serial molecular profiling would provide valuable insights into tumor evolution and resistance mechanisms under therapeutic pressure. By bridging these gaps, these studies hold the potential to significantly improve clinical outcomes and offer new hope for patients with several challenging malignancies.

## DECLARATIONS

### Availability of data and materials

The datasets presented in this study are available in the online repository cBioportal (https://www.cbioportal.org).

### Copyright

© The Author(s) 2025.
