# Supplementary material for: Unraveling BRAF alterations: molecular insights to circumvent therapeutic resistance across cancer types
Source: Cancer Drug Resist. 2025 Mar 24;8:14. doi: 10.20517/cdr.2024.213 (PMC11977354; doi:10.20517/cdr.2024.213)
Supplement: Supplementary file 1 [file cdr-8-14-SupplementaryMaterials.zip › Supplementary Materials-Annotation.pdf]

Supplementary Materials

Unraveling *BRAF* alterations: molecular insights to circumvent therapeutic resistance across cancer types

Caterina Perfetto[1,2,#], Marianna Aprile[1,#], Simona Cataldi[1], Elisa Giovannetti[3,4], Valerio Costa[1]

[1]Institute of Genetics and Biophysics (IGB), National Research Council of Italy (CNR), Naples 80131, Italy.
[2]Department of Environmental, Biological and Pharmaceutical Sciences and Technologies (DiSTABiF), University of Campania "Luigi Vanvitelli", Caserta, Italy.
[3]Department of Medical Oncology, Amsterdam UMC, VU University, Cancer Center Amsterdam, Amsterdam 1081 HV, The Netherlands.
[4]Fondazione Pisana per La Scienza, San Giuliano Terme 56017, Italy.
[#]Authors contributed equally.

Correspondence to: Dr. Valerio Costa, Institute of Genetics and Biophysics (IGB), National Research Council of Italy (CNR), Via P. Castellino 111, Naples 80131, Italy. E-mail: valerio.costa@igb.cnr.it; Prof. Elisa Giovannetti, Department of Medical Oncology, Amsterdam UMC, VU University, Cancer Center Amsterdam, De Boelelaan 1117, Amsterdam 1081 HV, The Netherlands. E-mail: e.giovannetti@amsterdamumc.nl

Supplementary File 1. List of tumor types for cohort #1 (curated cBioPortal data sets) and cohort #2 (GENIE v13).

Supplementary File 2. Count of all *BRAF* alterations (point mutations, deletions/amplifications and structural variants) in cohort #1 and cohort #2.

Supplementary File 3. Association analysis outcomes of clinical parameters and molecular features in CRC patients carrying *BRAF* point mutations.

Supplementary File 4. Association analysis outcomes of clinical parameters and molecular features in NSCLC patients carrying *BRAF* somatic mutations.

Supplementary File 5. Differentially expressed genes between CRC "*BRAF*-altered" *vs* "*BRAF*-unaltered".

Supplementary Figure 1. Analysis of mutational concurrence in PTC, ATC and melanoma. Mutation incidence diagrams in A) PTC, B) ATC, and C) melanoma, considering two different cohorts. In each box, the red color intensity is proportional to the mutation frequency of the gene.
